# The role of novel forest ecosystems in the conservation of wood‐inhabiting fungi in boreal broadleaved forests

**DOI:** 10.1002/ece3.2384

**Published:** 2016-09-07

**Authors:** Katja Juutilainen, Mikko Mönkkönen, Heikki Kotiranta, Panu Halme

**Affiliations:** ^1^ Department of Biological and Environmental Sciences University of Jyvaskyla PO Box 35 FI‐40014 Jyvaskyla Finland; ^2^ Biodiversity Unit Finnish Environment Institute PO Box 140 FI‐00251 Helsinki Finland; ^3^ Jyvaskyla University Museum University of Jyvaskyla PO Box 35 FI‐40014 Jyvaskyla Finland

**Keywords:** Afforested fields, corticioids, deadwood, fungal communities, natural herb‐rich forests, novel ecosystems, wood pastures

## Abstract

The increasing human impact on the earth's biosphere is inflicting changes at all spatial scales. As well as deterioration and fragmentation of natural biological systems, these changes also led to other, unprecedented effects and emergence of novel habitats. In boreal zone, intensive forest management has negatively impacted a multitude of deadwood‐associated species. This is especially alarming given the important role wood‐inhabiting fungi have in the natural decay processes. In the boreal zone, natural broad‐leaved‐dominated, herb‐rich forests are threatened habitats which have high wood‐inhabiting fungal species richness. Fungal diversity in other broadleaved forest habitat types is poorly known. Traditional wood pastures and man‐made afforested fields are novel habitats that could potentially be important for wood‐inhabiting fungi. This study compares species richness and fungal community composition across the aforementioned habitat types, based on data collected for wood‐inhabiting fungi occupying all deadwood diameter fractions. Corticioid and polyporoid fungi were surveyed from 67 130 deadwood particles in four natural herb‐rich forests, four birch‐dominated wood pastures, and four birch‐dominated afforested field sites in central Finland. As predicted, natural herb‐rich forests were the most species‐rich habitat. However, afforested fields also had considerably higher overall species richness than wood pastures. Many rare or rarely collected species were detected in each forest type. Finally, fungal community composition showed some divergence not only among the different habitat types, but also among deadwood diameter fractions. *Synthesis and applications*: In order to maintain biodiversity at both local and regional scales, conserving threatened natural habitat types and managing traditional landscapes is essential. Man‐made secondary woody habitats could provide the necessary resources and serve as surrogate habitats for many broadleaved deadwood‐associated species, and thus complement the existing conservation network of natural forests.

## Introduction

The biosphere of the earth is becoming increasingly transformed by human actions (Ellis and Ramankutty 2008). Changes in ecosystems include the loss of biodiversity, the invasion of nonnative species, altered natural disturbance dynamics, and biotic homogenization. As a consequence, human impact has resulted in the development of biological systems that differ in species composition or ecological function from historical and present systems – the so‐called novel ecosystems (Hobbs et al. 2006, 2009, 2013; Truitt et al. 2015). The management of these novel ecosystems should aim to maintain genetic and species diversity, and to promote healthy ecosystem function and services (Seastedt et al. 2008; Navarro and Pereira 2012).

At the landscape level, human interference manifests as loss, deterioration, and fragmentation of natural habitat types. In the boreal zone, the forest habitats have undergone the most radical changes: Previously continuous canopy cover has turned into separate forest patches surrounded by continuously extending urban and agricultural areas. Additionally, intensive forest management has considerably reduced both the amount and quality of deadwood in the forested landscape, jeopardizing countless deadwood‐dependent organisms (Siitonen 2001). Wood‐inhabiting fungi can be considered the most important deadwood‐associated species group, being greatly responsible for the decay processes (Boddy et al. 2008; Stokland et al. 2012). Earlier research on wood‐inhabiting fungi has mainly focused on the effects of forest management and on species associated with large‐diameter deadwood (i.e., coarse woody debris (CWD)) (Junninen and Komonen 2011). Nonetheless, it has been shown that many wood‐inhabiting fungi (also) utilize smaller deadwood particles (i.e., [very] fine woody debris ([V]FWD)) (Kruys and Jonsson 1999; Heilmann‐Clausen and Christensen 2004; Norden et al. 2004; Kueffer et al. 2008; Juutilainen et al. 2011; Abrego and Salcedo 2013). It has also been shown that each deadwood diameter fraction in coniferous forests hosts a partly unique, partially overlapping, fungal assembly (Juutilainen et al. 2014).

As the boreal forest region is dominated by coniferous forests, broadleaved forests are less common and limited in size. Due to their naturally fertile soil, large areas of natural herb‐rich forests had been widely converted into agricultural land already in the past. Thus, very few natural herb‐rich forest stands remain today, and most of them are small in size. Nowadays, they are recognized as threatened habitat types and are often protected. Natural herb‐rich forests are known to be species‐rich habitats for wood‐inhabiting fungi, as well as for many other taxonomical groups (Tonteri et al. 2008).

In addition to natural herb‐rich sites, there are several (partly) man‐made forest types which are dominated by broadleaved trees due to different reasons. On the one hand, wood pastures are seminatural, often broadleaved‐dominated woody habitats, which have been created for, and are maintained by, animal grazing. The semiopen, sparse canopy layer of wood pastures allows more sunshine to reach the forest floor than a closed forest cover. As a consequence, temperature of the ground layer and of the soil is higher. Moreover, animal grazing inflicts mechanical disturbance on the vegetation and the soil, while urine and dung deposits create local nutrient enrichment (Bergmeier et al. 2010). Wood pastures with a long grazing history have diverse vascular plant (Pykälä 2003; Pöyry et al. 2006) and ground fungi (Mustola 2012) communities. Due to the intensification of agriculture and the abandonment of traditional land use methods, wood pastures are currently also a scarce and threatened habitat (Schulman et al. 2008).

Afforested fields, on the other hand, are novel, man‐made woody habitats (Cramer et al. 2008; Navarro and Pereira 2012). The long history of agricultural practices has generated some unique characteristics. Due to continuous fertilization, the soil of afforested fields resembles the naturally fertile soil of herb‐rich forests. The canopy layer, however, is usually a monoculture of a certain tree species, while the understory vegetation can have idiosyncratic assemblies depending on the site characteristics, the preceding agricultural history, and macroclimate (Wall and Hytönen 2005). The importance of afforested fields for biodiversity is poorly known (Bremer and Farley 2010; Carson et al. 2010; Skłodowski 2014). To our knowledge, in the boreal zone, only Komonen et al. (2015, 2016) have studied biodiversity in afforested fields. Their results indicate that afforested fields host diverse ground‐inhabiting fungi as well as insect communities. However, the potential importance of afforested fields for wood‐inhabiting biota remains unknown.

The objective of this study was to compare saproxylic fungi among three woody habitat types: (1) natural herb‐rich forests, (2) wood pastures, and (3) afforested fields. Natural herb‐rich forests are known to host diverse communities of wood‐inhabiting fungi and of many other taxa. However, saproxylic fungal diversity associated with small‐diameter deadwood in natural herb‐rich forests is still poorly known. Additionally, to our knowledge, no systematic research on wood‐inhabiting fungi has been conducted in wood pastures or afforested fields. Thus, even the very basic information on the identity and ecology of saproxylic fungi occurring in these two habitat types is lacking. This study aimed to answer the following questions: (1) Do the wood‐inhabiting fungal species richness and community composition vary across the three woody habitat types? (2) Do natural herb‐rich forests, wood pastures, and afforested fields host special and noteworthy wood‐inhabiting fungal species, especially on FWD? (3) Can these novel habitats serve as surrogate habitats for broadleaved associated saproxylic fungal species?

## Material and Methods

### Study area

The study was conducted in central Finland, in the south and middle boreal vegetation zone (Ahti et al. 1968). Natural herb‐rich forests consist of a variable assembly of broadleaved tree species (see Hämet‐Ahti et al. 1998, for naming authorities), including birches *Betula* spp. (referred to as “birch” hereafter), *Populus tremula*,* Alnus incana*,* A. glutinosa*,* Sorbus aucuparia*,* Salix caprea*,* Prunus padus*,* Acer platanoides*,* Tilia cordata*, and *Ulmus glabra*, mixed with occasional *Picea abies* and *Pinus sylvestris* (see photograph of typical site, Fig. S1a). All of the studied natural herb‐rich forest sites are situated in nature reserves that belong to the Natura 2000 network.

Wood pastures are birch‐dominated, with variable amount of *Juniperus communis*,* A. incana, S. aucuparia, P. abies*, and *P. sylvestris* (see photograph of typical site, Fig. S1b). The sites are still in use for cattle or sheep grazing, and have total grazing history of around 100–200 years. All wood pasture sites are on privately owned land.

Afforested fields are almost completely birch‐dominated, with only occasional *Salix spp*. and *P. abies* saplings (see photograph of typical site, Fig. S1c). The fields were previously used for grain and hay farming and were repeatedly fertilized. These sites were afforested for birch during 1990–1992 as a part of Finnish Forest Research Institute's (Metla) field afforestation experiment (Ferm et al. 1993). All afforested field sites are on privately owned land.

### Study design and sampling methods

Each habitat type was represented by four analogous sites. Three replicate 10 × 10 m sampling plots were established at each natural herb‐rich forest and wood pasture study site (12 plots in total) according to the methodology used in Juutilainen et al. (2011, 2014). More details about the wood pasture sites and the vegetation in the sampling plots can be found in Oldén et al. (2016). Due to extremely laborious data collection in afforested field sites, and as only two 10 × 10 m sample plots could be fitted inside one birch afforestation plot, the number of sampling plots per site was reduced to two (thus, 8 plots in total). Same afforested field sites have also been used to investigate insect fauna and ground fungi (Komonen et al. 2015, 2016). At every corner of a sampling plot, we placed a 2 × 2 m subplot. In wood pastures and natural herb‐rich forests, combined subplot area equals to 192 m² (0.0192 ha) and total area of sampling plots was 1200 m² (0.12 ha), and in afforested fields 128 m² (0.0128 ha) and 800 m² (0.08 ha), respectively.

From each subplot, all observed deadwood particles (excluding leaves, needles, litter, and herbaceous plant stems) were recorded and examined and the proximal diameter of each particle was estimated. Outside the subplots, in the remaining sample plot area, deadwood particles with a minimum diameter of 2 cm were recorded and examined. Deadwood particles were divided into six diameter categories: <0.5, 0.5–<1, 1–<2, 2–<5, 5–<10, and ≥10 cm. These limiting values were chosen in order to have comparable data to those from previous studies on coniferous forests (Juutilainen et al. 2011, 2014). Deadwood particles were identified to species level when possible. In natural herb‐rich sites, especially with more decayed particles, this was often impossible. Hence, many deadwood particles were labeled as “Unidentified hardwood.”

Within the sample plots, all deadwood particles (including logs, snags, stumps, branches, and twigs) were carefully examined for the presence of fruit bodies of wood‐inhabiting fungi. Living trees were examined more superficially. This study focused on corticioid and polyporoid fungi (Aphyllophorales, Basidiomycota), including resupinate Heterobasidiomycetes (Corticiaceae *s.l*.). The abundance of each species was recorded as the number of deadwood particles on which it was found. Surveyed fungi were identified to species level whenever possible. The specimens were identified *in situ* or collected and dried for later microscopic identification. A compound microscope with magnification of 40–1600× was used for identification. The nomenclature is not based on any one particular manual, but it follows that in Kotiranta et al. (2009), with some exceptions from Bernicchia and Gorjón (2010) and Ryvarden and Melo (2014). Voucher specimens are preserved in the herbarium of Natural History Museum of University of Jyväskylä (JYV) and in the personal collections of the authors (K.J. and H.K). In this study, “rarely observed species” are considered having less than 10 earlier collections from Finland according to Kotiranta et al. (2010), Kunttu et al. (2011, 2012, 2013, 2014), and H. Kotiranta (update 2015, personal communication). Red‐listed species and the threat categories are awarded according to Kotiranta et al. (2010). A complete list of all taxa found in this study can be found in Appendix S1. Fieldwork for natural herb‐rich forest sites was conducted between 24 August and 25 October 2007. The wood pastures and two afforested field sites were surveyed in 2012 between 13 September and 5 November, and the other two afforested field sites between 9 September and 5 October 2013. The timing coincides with peak fruiting season of wood‐inhabiting fungi in the study area (Halme and Kotiaho 2012).

### Data analyses

The effect of forest type, substrate diameter category, and study site, as well as their interactions, on the number of fungal species was analyzed with generalized linear models (GENLIN) multivariate procedure. As the number of species and occurrences is count data, a Poisson log‐linear model type was selected. In the model, the number of fungal occurrences was assigned as covariate; forest type, substrate diameter category, and study site were assigned as fixed factors. In order to take into account the nested study design (in which the sample plots are within the sites and the sites within the forest types), two nested terms were built. For estimation of parameter values, Pearson chi‐square‐based scale parameter method was selected. To test model effects, type III analysis was used as it calculates adjusted sums of squares for both main effects and interactions, and thus is best suited for unbalanced or nonorthogonal data (Hector et al. 2010). The nonparametric test statistics were selected because of large number of zeros and inequality of variances among the classes in the data. IBM SPSS Statistics 22.0 software for Windows was used for the analyses (IBM Corp, 1 New Orchard Road, Armonk, NY).

To compare total species richness among forest types, species accumulation curves for each forest type were constructed using sample‐based rarefaction (Gotelli and Colwell 2001; Magurran 2004). The elevation of the curves indicates differences in the number of detected species among sample units. Curvature and leveling of the slope reveals the likelihood of undetected species and the effectivity of sampling effort. Rarefaction was calculated using study plots as sample units. The sample size for natural herb‐rich forests and wood pastures was 12 (four sites in both forest types and three sampling plots in every site), but for afforested fields eight (four sites with two sampling plots in each). Therefore, the sampling size for all forest types was extrapolated to 16 sites (Colwell et al. 2012). This is double the smallest reference sample size, which according to Chao et al. (2014) is the maximum reasonable sample size for extrapolated sample‐based species accumulation curves. Because the coefficient of variation of the abundance distribution was >0.5 in each forest type dataset, the classic formula of Chao1 richness estimator was used. Species richness (*S*_est_) and 95% confidence intervals were calculated using EstimateS 9.1.0 software for Windows (Colwell 2013).

The species assembly of different forest types was compared with nonmetric multidimensional scaling (NMS; see McCune and Grace 2002; for a summary and references), using Sørensen distance measure. A response matrix was constructed using diameter class‐specific species abundance data from every forest site (six diameter classes, 12 sites). The sample plots within each forest site were pooled together (three plots per site for natural herb‐rich forests and wood pastures; two plots per site for afforested fields). The two largest diameter categories were omitted from one afforested field site, and the largest category from another afforested field site because of zero occurrences. Species‐ and genus‐level fungal observations were included in the analyses (here referred to as “species”). The final response matrix included 209 species and 69 sample units. To select appropriate dimensionality, two autopilot test runs were conducted for 1–6 dimensions (250 runs with real and 250 with randomized data). Stress reduction was determined after 500 iterations using Monte Carlo simulation, after which a three‐dimensional solution was recommended (final stress 21.77; final instability 0.00097; *P* = 0.04). However, the stress level was rather high in every solution, and a stability criterion was not met. Generally, ordination axes with stress values <10 are considered reliable and readily interpretable, and values over 30 indicate little correspondence with the original data. Thus, with stress values around 20, the ordinations can still be useful, but better interpreted only at a broad scale and indicative of general trends (Clarke 1993). Finally, five runs with three‐dimensional solution were made (each with 250 runs with real and 249 runs with randomized data; 250 iterations). Varimax rotation was used in order to have better comparable axes among the runs. The 2nd run gave the most visually applicable picture, and was thus selected for further use (minimum stress 21.749; *P* = 0.004). Differences among the forest types and substrate diameter categories were further tested with ranked multiresponse permutation procedure (MRPP). Ordination and permutation analyses were performed with PC‐ORD 5.33 (McCune and Mefford 2006).

## Results

**Figure 1:**
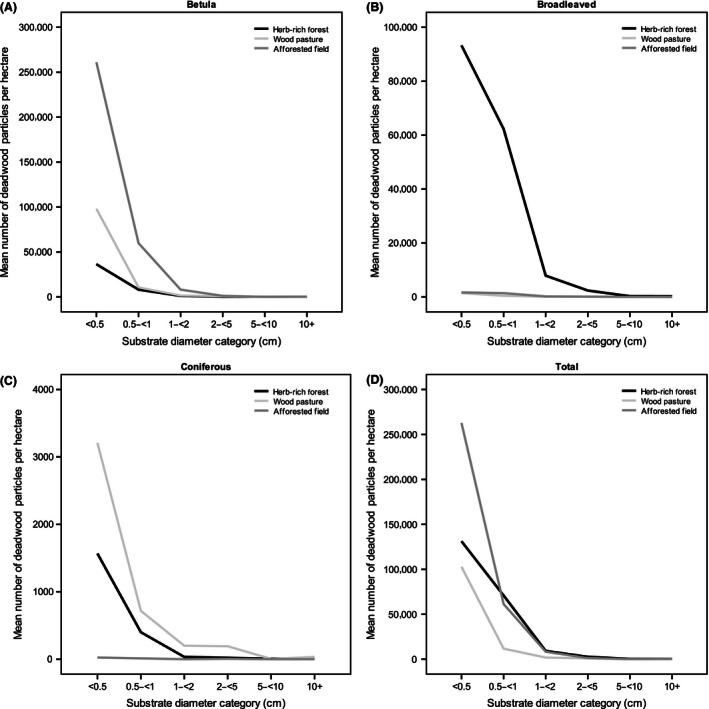
The relationship between the mean number (per hectare) of deadwood particles and substrate diameter category across three forest types: (A) birch (*Betula* spp.), (B) combined deciduous, (C) combined coniferous, and (D) all combined deadwood. Note the different scaling for *y*‐axis in respective figures.

In total, 67,130 deadwood particles were examined for fungi presence. The total number of deadwood particles was highest in the afforested fields and was comprised almost entirely of birch wood. Natural herb‐rich forests contained the highest number of other broadleaved deadwood. Wood pastures had more mixed‐in coniferous deadwood than the other habitat types. The amount of VFWD was remarkably high in afforested fields. CWD in natural herb‐rich forests consists of logs and snags, while in wood pastures and afforested fields, CWD is mainly stumps. Also, the amount of CWD was clearly higher in natural herb‐rich forests than in the other habitat types (Table 1; Fig. 1).

**Table 1:** Mean number (and standard deviation) of deadwood particles (birches, other broadleaved wood combined, and coniferous wood combined) recorded per hectare for each forest type and diameter fraction. For the three largest diameter categories, the values for stumps are given separately to those from other deadwood types

	Wood Pasture	Herb‐rich Forest	Afforested Field
*Betula* spp.			
<0.5	98,075.0 (20,040.7)	36,391.7 (26,822.3)	261,100.0 (86,645.5)
0.5–<1	10,525.0 (1514.0)	8100.0 (5656.0)	59,837.5 (13,040.0)
1–<2	1541.7 (281.3)	1166.7 (501.7)	8087.5 (1779.0)
2–<5	1191.7 (528.3)	333.3 (239.3)	2212.5 (1293.5)
2–<5 stumps	33.3 (40.7)	0	75.0 (103.0)
5–<10	33.3 (33.3)	125.0 (130.0)	150.0 (203.0)
5–<10 stumps	0	0	37.5 (65.0)
10+	41.7 (36.3)	125.0 (118.7)	0
10+ stumps	33.3 (40.7)	0	475.0 (476.5)
Broadleaved combined			
<0.5	1425.0 (1400.3)	93,258.3 (53,278.3)	1662.5 (2624.5)
0.5–<1	416.7 (440.0)	62,300.0 (29,871.7)	1400.0 (2143.5)
1–<2	75.0 (76.0)	7908.3 (3158.3)	200.0 (226.5)
2–<5	75.0 (54.7)	4758.3 (1502.3)	162.5 (281.5)
2–<5 stumps	41.7 (43.3)	0	87.5 (151.5)
5–<10	16.7 (16.7)	558.3 (541.3)	25.0 (25.0)
5–<10 stumps	0	0	12.5 (21.5)
10+	8.3 (14.3)	491.7 (272.3)	0
10+ stumps	0	16.7 (29.0)	0
Coniferous combined			
<0.5	3208.3 (1902.7)	1566.7 (1559.3)	25.0 (43.5)
0.5–<1	716.7 (378.3)	400.0 (455.3)	12.5 (21.5)
1–<2	200.0 (113.0)	33.3 (23.7)	
2–<5	291.7 (103.7)	41.7 (27.7)	12.5 (21.5)
2–<5 stumps	91.7 (98.3)	0	0
5–<10	8.3 (14.3)	16.7 (16.7)	0
5–<10 stumps	0	0	0
10+	0	41.7 (43.3)	0
10+ stumps	66.7 (70.7)	0	0
Total			
<0.5	102,708.3 (23,343.3)	131,216.7 (81,660.0)	262,787.5 (89,313.0)
0.5–<1	11,658.3 (2332.3)	70,800.0 (35,983.0)	61,250.0 (15,205.0)
1–<2	1816.7 (686.7)	9108.3 (3683.7)	8287.5 (2005.0)
2–<5	1558.3 (686.7)	5133.3 (1769.0)	2387.5 (1596.5)
2–<5 stumps	116.7 (182.3)	0	162.5 (254.5)
5–<10	58.3 (64.3)	700.0 (688.0)	175.0 (228.0)
5–<10 stumps	0	0	50.0 (86.5)
10+	50.0 (50.7)	658.3 (434.3)	0
10+ stumps	100.0 (111.7)	16.7 (29.0)	475.0 (476.5)

Of the inspected deadwood particles, 90% were apparently empty, meaning there were no visible fruiting bodies or fungal cords present. Altogether, 7036 fungal observations were made from 6692 deadwood particles. More than one taxon was recorded from 344 deadwood particles. A total of 3925 observations were of sterile fruit bodies, anamorphs, or cords and remained therefore unidentified to higher taxonomic level. A total of 2538 records of 194 species‐level and 573 genus‐level observations were used in the analyses (Appendix S1).

Comparing the different habitat types, natural herb‐rich forests had the highest overall species richness (136 species, 1061 observations), whereas afforested fields yielded the most observations (98 species, 4959 observations). The survey of wood pastures resulted in 79 species and 1016 observations. The sample size from afforested fields was notably high, as the observations originate from 33% smaller surface area (80 m²) than those from natural herb‐rich forests and wood pastures (120 m² each).

Unique species were recorded in every habitat type: 60 species were found only in natural herb‐rich forests, 28 species only in afforested fields, and 23 species only in wood pastures. In addition, each deadwood diameter fraction contained several species absent from other fractions: The smallest diameter fraction (<0.5 cm) contained 13 unique species; the 0.5–<1 cm fraction contained five unique species; the 1–<2 cm fraction contained 11 unique species; the 2–<5 cm fraction contained 27 unique species; the 5‐<10 cm fraction contained nine unique species; and the 10+ cm fraction contained 18 unique species.

In total, 23 rare or rarely collected species (with <10 previous observations from Finland) were detected, ten of which were found only from natural herb‐rich forests, seven only from afforested fields, and four only from wood pastures. Two rare species were found from both natural herb‐rich forests and afforested fields. Also, four red‐listed species were collected, two of which from natural herb‐rich forests, one from wood pastures, and one from afforested fields. Furthermore, three species new to Finland (*Cristinia rheana* from wood pasture site, *Hyphodontiella hauerslevii* from natural herb‐rich forest and afforested field site, and *Xenasma pruinosum* from two natural herb‐rich forest sites) and several probably yet undescribed species were collected (Appendix S1).

**Figure 2:**
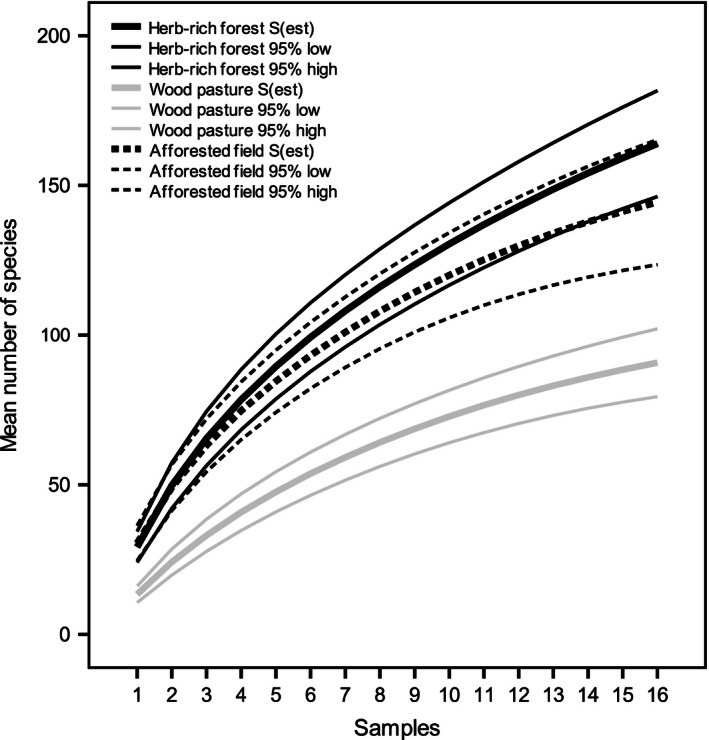
Observed species accumulation curves across forest types. The higher and lower 95% confidence intervals are represented by thinner lines. For herb‐rich forests and wood pastures, samples 1–12 represent real data and samples 12–16 are extrapolated from complete substrate data. For afforested fields, samples 1–8 represent real data and the samples 9–16 are extrapolated.

According to sample‐based rarefaction, species richness was highest in natural herb‐rich forests, and almost as high in afforested fields (Fig. 2). The difference is clearer at the higher levels of sampling effort; at the lowest levels (the first two samples), the curves were almost identical. In contrast, the species richness of wood pastures was notably lower. The nonoverlap of 95% confidence intervals can be interpreted as a statistically significant difference in species richness (Payton et al. 2003). None of the species accumulation curves showed signs of leveling toward an asymptote. This indicates that despite our laborious sampling effort, the collected data do not comprehensively represent the background fungal community. However, the species accumulation curves for wood pastures and afforested fields were more gently sloping than the curve for natural herb‐rich forests, suggesting that a more complete sample of the fungal community was obtained in these forest types.

**Figure 3:**
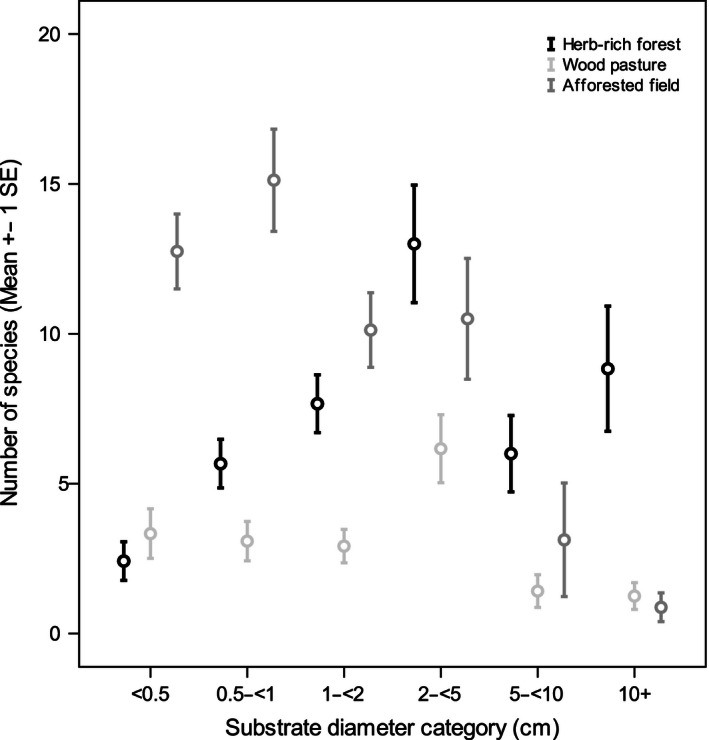
Mean number of fungal species (±SE) observed for each forest type and substrate diameter category.

Habitat type, substrate diameter category, and study site affected the number of detected fungal species, and a significant interaction effect between habitat type and substrate diameter was also found (Table 2; Fig. 3). Also, the number of species increased as the number of fungal observations increased. In contrast, individual study plots had no significant effect on species richness. The significant interaction between habitat type and substrate diameter category reflects the difference in species richness of different substrate diameter categories among the habitat types. Natural herb‐rich forests contained highest species richness in the medium and large deadwood fractions. In afforested fields, the smallest diameter fractions were the most species‐rich. Species richness in wood pastures was rather low over all deadwood fractions, with a peak in the medium‐sized deadwood. All pairwise comparisons among the forest types were significant (herb‐rich forest vs. wood pasture, mean difference = 3.81, SE = 0.525, *P* < 0.001; herb‐rich forest vs. afforested field, mean difference = 1.97, SE = 0.725, *P *= 0.006; wood pasture vs. afforested field, mean difference = 1.84, SE = 0.668, *P* = 0.006).

**Table 2:** The effects of different explaining variables on the number of detected fungal species in the study sites (Generalized Linear Model)

Dependent variable	Wald chi‐square	df	*P*
Intercept	307.669	1	<0.001
Study plot (within Site)	10.633	8	0.223
Site (within Forest type)	51.132	6	<0.001
Forest type	47.413	2	<0.001
Diameter category	80.410	5	<0.001
Number of observations	28.842	1	<0.001
Forest type * Diameter category	46.599	10	<0.001

**Figure 4:**
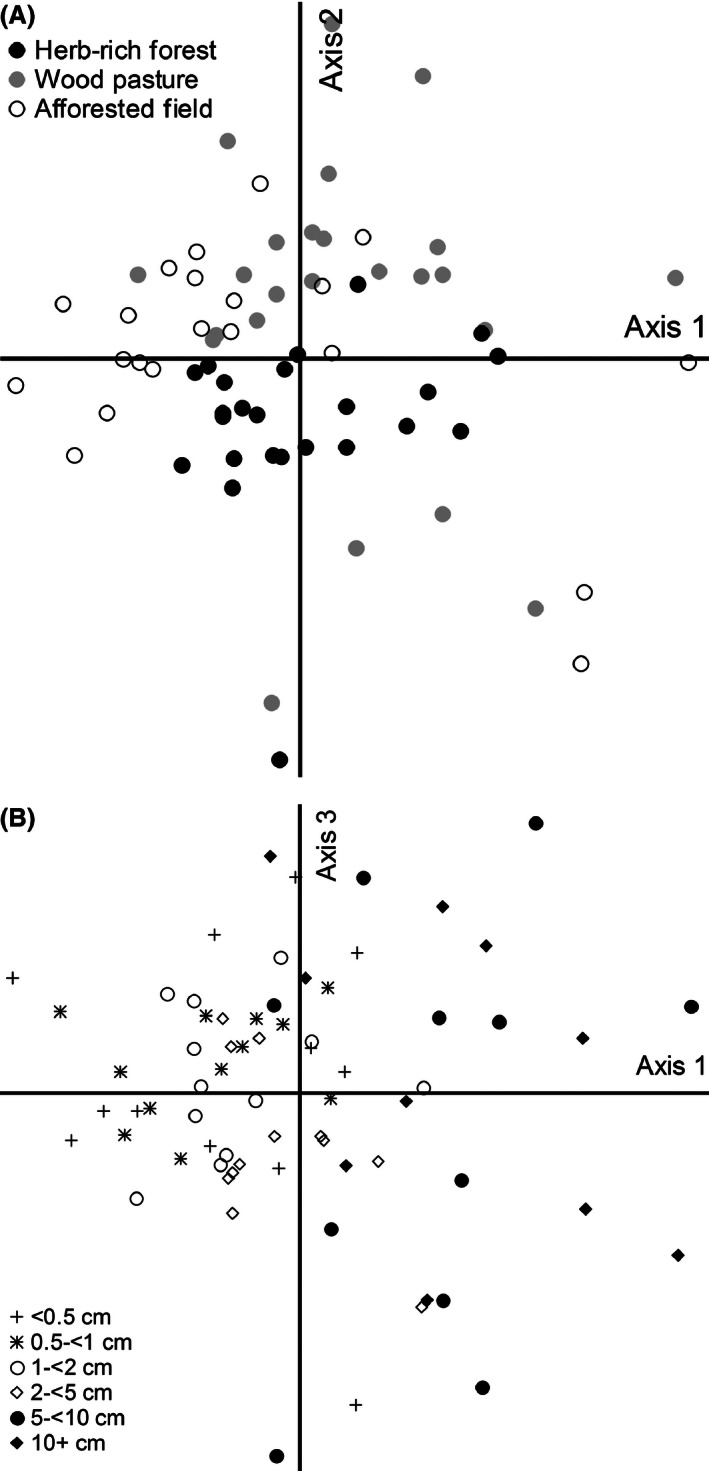
Two‐dimensional NMS‐ordination plots of fungal communities representing 69 sites divided by (A) habitat type and (B) substrate diameter category.

The three‐dimensional ordination space produced by NMS explained 58% of the variation in fungal assemblages across study sites. Axis 1 represented 25%, axis 2 represented 20%, and axis 3 represented 13% of the variance, respectively. We can interpret two patterns from the ordination space results. First, axis 2 divided wood pastures and afforested fields (positive side of the center; Fig. 4A) from natural herb‐rich forests (negative side). Second, different deadwood diameter fractions distributed from the smallest to the largest along axis 1 and formed loose, partly overlapping groups (Fig. 4B). The two smallest diameter fractions are positioned near the negative end of axis 1, while the middle‐sized fractions tended to concentrate more around the center. The two largest diameter fractions were scattered around the positive side of axis 1. The observed visual patterns were further supported by the MRPP tests. The differences in fungal community composition among all forest types were significant (*T* = −10.67, *A* = 0.116, *P* < 0.001; pairwise comparisons in Table S1). The fungal community composition did not differ significantly among every substrate diameter category pair, but the division of fungal communities among VFWD/FWD/CWD was clear (*T* = −6.97, *A* = 0.122, *P* < 0.001; pairwise comparisons in Table S1). In both tests, *A* >0 indicating that there is less heterogeneity within groups than expected by chance; that is, the habitat types and substrate diameter classes are clearly distinguishable.

## Discussion

According to our results, deadwood‐associated fungal species richness was highest in natural herb‐rich forests, especially in medium‐ and large‐diameter deadwood fractions. This is not surprising as the high overall biodiversity of natural herb‐rich forests (Tonteri et al. 2008) and deciduous CWD (Heilmann‐Clausen and Christensen 2004; Norden et al. 2004; Markkanen and Halme 2012; Abrego and Salcedo 2013) is well established. However, we found that fungal species richness in small‐diameter deadwood was highest in afforested fields. The latter can be partially explained by the large quantity of small‐diameter deadwood readily available (Table 1). For “non‐resource‐unit‐restricted” saproxylic fungi, this means short distance for mycelial dispersal between deadwood pieces, and therefore enables cost‐effective transition into a new resource patch (Boddy et al. 2008). Inversely, the lower fungal species richness of wood pastures may stem from scarcity of suitable resources: The rather open canopy in most wood pastures would result in smaller and often localized deadwood patches. Additionally, animal grazing keeps the ground vegetation low, which, combined with moderate exposure to sun and wind, may result in drier local microclimate that discourages fungal growth.

Species abundance, in turn, was notably higher in afforested fields than in the other two habitat types. The number of fungal observations was almost five times higher in afforested fields, even though it originated from a smaller surface area sampled. On the other hand, the number of observations in natural herb‐rich forests and wood pastures was almost equal, even if the species richness was considerably higher in herb‐rich forests. The number of individuals and species in afforested fields was higher than we expected, especially as practically all of them derive solely from birch wood. Such man‐made habitats are new, but perhaps are somewhat similar to those present during the era of *slash‐and‐burn* cultivation, which was still a common practice in the beginning of the 20th century. After burning, the fields were cultivated a few years and then abandoned as nutrients were depleted from the soil. Following abandonment, the vegetation recovered, with the most common trees on these fields being Scotch pine, birches, and gray alder. Sometimes, birch was dominant or even the only tree species present, and these areas would cover thousands of hectares (Heikinheimo 1915). Unfortunately, when these birch forests covered large areas in central Finland, there were no mycologists collecting data from such habitats. Therefore, we cannot make any comparisons of the species assemblages between these two kinds of birch forests. It is possible, however, that *Phlebiella tulasnelloidea*, which is rarely collected in Finland, was common on those succession forests post‐slash‐and‐burn cultivation.

Unique fungal species were found in every habitat type, and their numbers reflect the overall species richness of the corresponding habitat types. Over twice as many unique species were detected in natural herb‐rich forests compared with the other two habitat types. This corroborates the importance of natural habitats, and their diverse microhabitats within, for broadleaved deadwood‐associated fungi. In addition, several rare or rarely encountered fungal species were recorded in every habitat type. The number of these species is high, but not overly surprising. Most observations were made from <0.5‐cm‐diameter deadwood, while the other substrate diameter fractions were represented more equally. Any comprehensive sampling from little‐studied resources and habitats is likely to produce species which are new to (*Cristinia rheana* and *Hyphodontiella hauerslevii*) or very seldom collected in (*Phlebiella insperata, Ramaricium alboochraceum, Sebacina helvelloides*, and *Sistotrema autumnale*) Finland. A common feature to almost all of these taxa is that the basidiocarps are very tiny and therefore easily overlooked. There are, however, some species with easily observable basidiocarps (e.g., *Byssomerulius jose‐ferreirae*) that are surely rare.

The current knowledge of the ecology and habitat requirements of many deadwood‐associated fungal species is still very limited. In particular, the identity of species occupying (V)FWD is poorly known. As we discussed in our previous study of coniferous forests (Juutilainen et al. 2014), many species that are considered rare are, in fact, only rare on CWD. Therefore, their classification as rare merely reflects the biased sampling effort toward CWD and polypores. We assume that many so‐called rare broadleaved deadwood‐associated species may actually be numerous throughout forested landscape. However, tests of this hypothesis require a more comprehensive and systematic survey of broadleaved woody habitats.

Species accumulation curves further highlighted the differences in fungal species richness among the three habitat types. Lower species richness in wood pastures was evident already at low sampling effort. However, the curves for natural herb‐rich forests and afforested fields overlapped at low sampling effort. Only as the sample size increased, did the difference between the two curves become more evident. However, even with extrapolated data, the 95% confidence intervals were still overlapping. Furthermore, none of the curves showed signs of leveling off, suggesting that despite our considerable sampling effort of almost 70,000 deadwood particles, several species were undetected in the fruiting fungal communities in each habitat type. This is particularly true for natural herb‐rich forests that show a steeper (cf. gently rising) curve. Therefore, to reveal the real difference in species richness among the different habitat types, a very high sampling effort is needed. The latter must also include all deadwood diameter fractions in order to achieve a comprehensive picture of fungal communities. Also, it is widely agreed that inventories based solely on the presence of fruit bodies cannot describe the whole community, as many fungal species are only present as mycelia at given period of time (Boddy et al. 2008; van der Linde et al. 2012; Ovaskainen et al. 2013). Therefore, if the objective is to identify the members of a whole fungal community as accurately as possible, a thorough comprehensive approach combining fruit body and mycelial surveys is necessary (Halme et al. 2012).

Our results indicate that habitat type and diameter of deadwood substrate are very important factors affecting fungal community composition. The differences in deadwood profiles among the habitat types can be considered to be the main source of variation in the local species pool. The high qualitative diversity of deadwood substrates in natural herb‐rich forests creates multiple ecological niches for individual fungal species to occupy. The natural forest structure and dynamics provide, in turn, variable microclimatic conditions to meet the demands of even the more demanding wood‐inhabitants. The deadwood substrates originating from afforested fields are comprised of very small‐diameter birch twigs and branches that occur in large quantities, and thus provide suitable habitat for a more limited assembly of saproxylic fungi. In wood pastures, the scarcity of variable deadwood resources, together with more exposed environmental conditions, confines the potential species assembly even further and favors generalist species. In addition, study site identity had a significant effect, which implies there is intrinsic variation at site level, stemming from local environmental conditions and/or stochastic events. As the individual study plots had no significant effect on fungal species richness or assembly, local metapopulation dynamics appear to operate at forest stand or habitat level. This result is in contradiction to the finding by Abrego et al. (2014), who showed that fungal community structure was already largely determined at the sample plot scale. The most likely reason for this difference is that in this study, the sample plots within each site were closer to each other than in Abrego et al.'s study. Moreover, the spatial scale of community turnover is naturally largely determined by the (scale of) natural patchiness of a given habitat.

The fungal diversity observed in afforested fields and wood pastures indicates that many wood‐inhabiting fungi can, indeed, survive and thrive in secondary woody habitats. Thus, afforested fields and wood pastures could serve as surrogate habitats for many broadleaved deadwood‐associated fungal species. Moreover, several rare or rarely collected fungi as well as new fungal species to Finland were recorded from both wood pastures and afforested fields, which suggests these secondary woody habitats have their own intrinsic value. However, in their current conditions, these man‐made habitats are so scarce in coarse woody debris that they cannot provide suitable habitats for a large part of wood‐inhabiting fungi. Therefore, adding wood pastures and afforested fields into existing herb‐rich forest conservation network would create important ecological corridors and stepping stones linking natural forest patches. Encouraging landowners to create and leave more CWD into wood pastures and afforested fields would make them more suitable habitats for a wider array of wood‐inhabiting fungi than that already present in their current state. By providing suitable substrates and microhabitats, these green corridors could facilitate mixing of separate fungal populations and enhance the survival probability of various species. As the coverage of natural forests is declining globally, ensuring sufficient amount and diversity of secondary woody habitats in nonforested landscape would provide a lifeline for many deadwood‐associated species.

## Conflict of Interest

None declared.

## Supporting information


**Table S1**. Full MRPP test results for forest types and substrate diameter categories including pairwise comparisons.

**Figure S1.** Photographic examples of study sites.

**Appendix S1.** List of species and higher taxonomic groups observed in the study.
