# Supplementary material for: The role of novel forest ecosystems in the conservation of wood‐inhabiting fungi in boreal broadleaved forests
Source: Ecol Evol. 2016 Sep 7;6(19):6943–54. doi: 10.1002/ece3.2384 (PMC5513230; doi:10.1002/ece3.2384)
Supplement: Supplementary file 1 — Table S1. Full MRPP test results for forest types and substrate diameter categories including pairwise comparisons. [file ECE3-6-6943-s001.docx]

**Table S1. Full ranked MRPP test results including pairwise comparisons for a) forest types and b) substrate diameter categories.**

1. Forest type

Overall test statistics: T= -10.67 A= 0.116 *P*< 0.001

| **Pairwise comparisons** | **T** | **A** | ***P*** |
| --- | --- | --- | --- |
|  |  |  |  |
| Herb-rich forest vs. Wood pasture | -8.43 | 0.10 | <0.001 |
| Herb-rich forest vs. Afforested field | -4.51 | 0.05 | <0.001 |
| Wood pasture vs. Afforested field | -9.82 | 0.11 | <0.001 |

1. Substrate diameter category

Overall test statistics: T= -6.97 A= 0.122 *P*< 0.001

| **Pairwise comparisons** | **T** | **A** | ***P*** |
| --- | --- | --- | --- |
|  |  |  |  |
| <0.5 cm vs. 0.5- <1 cm | 0.59 | -0.01 | 0.691 |
| <0.5 cm vs. 1- <2 cm | -1.37 | 0.03 | 0.093 |
| <0.5 cm vs. 2- <5 cm | -2.94 | 0.07 | 0.007 |
| <0.5 cm vs. 5- <10 cm | -3.37 | 0.08 | 0.002 |
| <0.5 cm vs. 10+ cm | -4.25 | 0.10 | <0.001 |
| 0.5- <1 cm vs. 1- <2 cm | 1.00 | -0.02 | 0.848 |
| 0.5- <1 cm vs. 2- <5 cm | -1.82 | 0.05 | 0.052 |
| 0.5- <1 cm vs. 5- <10 cm | -6.68 | 0.16 | <0.001 |
| 0.5- <1 cm vs. 10+ cm | -7.10 | 0.16 | <0.001 |
| 1- <2 cm vs. 2- <5 cm | -0.34 | 0.01 | 0.327 |
| 1- <2 cm vs. 5- <10 cm | -5.67 | 0.14 | <0.001 |
| 1- <2 cm vs. 10+ cm | -6.72 | 0.15 | <0.001 |
| 2- <5 cm vs. 5- <10 cm | -2.89 | 0.07 | 0.006 |
| 2- <5 cm vs. 10+ cm | -5.41 | 0.12 | <0.001 |
| 5- <10 cm vs. 10+ cm | 0.53 | -0.01 | 0.687 |
