# Supplementary material for: The role of novel forest ecosystems in the conservation of wood‐inhabiting fungi in boreal broadleaved forests
Source: Ecol Evol. 2016 Sep 7;6(19):6943–54. doi: 10.1002/ece3.2384 (PMC5513230; doi:10.1002/ece3.2384)

**Figure S1. Photographic examples of study sites: a) natural herb-rich forest, b) wood pasture, c) afforested field.**

a)


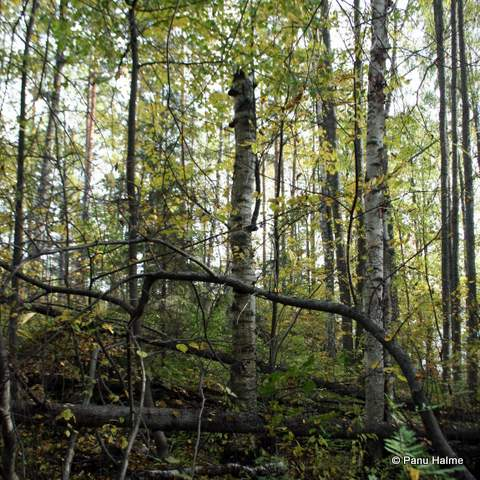


b)


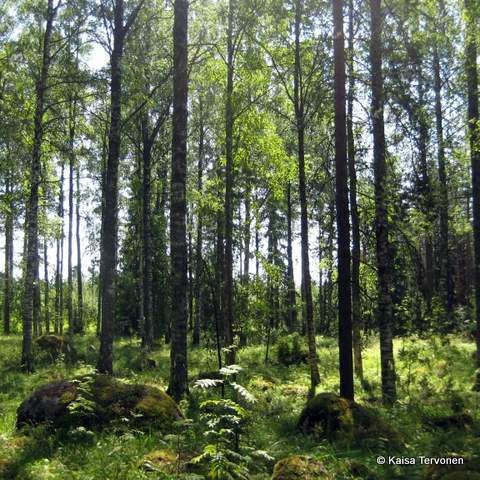


c)


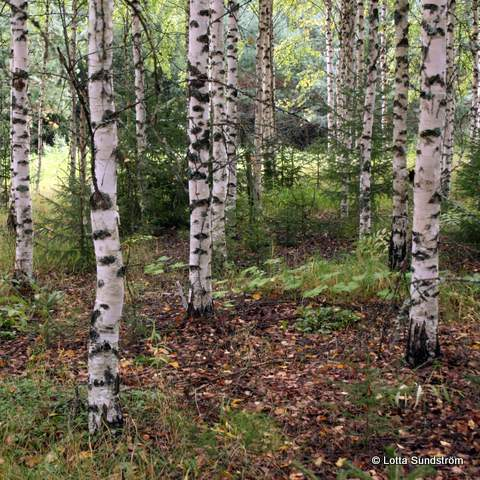

Supplement: Supplementary file 2 — Figure S1. Photographic examples of study sites. [file ECE3-6-6943-s002.docx]
