# Supplementary material for: The role of novel forest ecosystems in the conservation of wood‐inhabiting fungi in boreal broadleaved forests
Source: Ecol Evol. 2016 Sep 7;6(19):6943–54. doi: 10.1002/ece3.2384 (PMC5513230; doi:10.1002/ece3.2384)
Supplement: Supplementary file 3 — Appendix S1. List of species and higher taxonomic groups observed in the study. [file ECE3-6-6943-s003.docx]

**Appendix S1. List of species and higher taxonomic groups observed in the study.** The observations are divided according to forest type and substrate diameter category. Rarely observed species (less than 10 observations from Finland; (Kotiranta *et al.*, 2010; Kunttu *et al.*, 2011; Kunttu, Kulju, Kotiranta, 2012; Kunttu *et al.*, 2013; Kunttu *et al.*, 2014), updated situation in 2015 by Kotiranta, personal communication) are marked with asterisk (*). Three species new to Finland are marked with double asterisk (**). The nomenclature doesn’t follow any manual, but is mostly according to (Kotiranta, Saarenoksa, Kytövuori, 2009), with some exceptions from (Bernicchia, Gorjón, 2010) and (Ryvarden, Melo, 2014), where the naming authorities can be found.

|  | **Substrate diameter category ( 0= <0.5 cm, 1= 0.5-<1 cm, 2= 1-<2 cm, 3= 2-<5 cm, 4= 5-<10 cm, 5= 10+ cm)** | | | | | | | | | | | | | | | | | | | | | |  |  |  |  |  |  |  |
| --- | --- | --- | --- | --- | --- | --- | --- | --- | --- | --- | --- | --- | --- | --- | --- | --- | --- | --- | --- | --- | --- | --- | --- | --- | --- | --- | --- | --- | --- |
|  | **Herb-rich Forest** | | |  |  |  |  | **Wood Pasture** | | |  |  |  |  | **Afforested Field** | | |  |  |  |  | **Grand** |  |  |  |  |  |  |  |
|  | **Total** | **0** | **1** | **2** | **3** | **4** | **5** | **Total** | **0** | **1** | **2** | **3** | **4** | **5** | **Total** | **0** | **1** | **2** | **3** | **4** | **5** | **Total** |  |  |  |  |  |  |  |
| **Species** |  |  |  |  |  |  |  |  |  |  |  |  |  |  |  |  |  |  |  |  |  |  |  |  |  |  |  |  |  |
| *Amphinema byssoides* | **3** | 1 | 1 | 1 |  |  |  | **10** | 2 | 3 | 3 | 2 |  |  | **136** | 108 | 22 | 4 | 1 | 1 |  | **149** |  |  |  |  |  |  |  |
| *Amylostereum laevigatum* |  |  |  |  |  |  |  | **9** |  | 1 | 4 | 4 |  |  |  |  |  |  |  |  |  | **9** |  |  |  |  |  |  |  |
| *Amyloxenasma allantospora ** | **1** |  |  | 1 |  |  |  |  |  |  |  |  |  |  |  |  |  |  |  |  |  | **1** |  |  |  |  |  |  |  |
| *Antrodiella pallescens* | **2** |  |  |  |  |  | 2 |  |  |  |  |  |  |  |  |  |  |  |  |  |  | **2** |  |  |  |  |  |  |  |
| *Antrodiella romellii* | **7** |  |  | 4 | 3 |  |  | **3** |  | 1 | 1 | 1 |  |  | **24** |  | 7 | 9 | 8 |  |  | **34** |  |  |  |  |  |  |  |
| *Aphanobasidium cf. subnitens ** | **1** | 1 |  |  |  |  |  |  |  |  |  |  |  |  |  |  |  |  |  |  |  | **1** |  |  |  |  |  |  |  |
| *Athelia bombacina* |  |  |  |  |  |  |  |  |  |  |  |  |  |  | **1** | 1 |  |  |  |  |  | **1** |  |  |  |  |  |  |  |
| *Athelia decipiens* | **2** |  |  |  |  | 1 | 1 | **2** |  | 2 |  |  |  |  | **1** |  | 1 |  |  |  |  | **5** |  |  |  |  |  |  |  |
| *Athelia epiphylla* | **6** |  |  | 1 | 2 |  | 3 | **2** |  |  |  | 1 | 1 |  | **39** | 11 | 13 | 4 | 9 | 2 |  | **47** |  |  |  |  |  |  |  |
| *Athelia fibulata* |  |  |  |  |  |  |  | **11** | 5 | 1 | 2 | 3 |  |  | **31** | 11 | 6 | 5 | 6 | 3 |  | **42** |  |  |  |  |  |  |  |
| *Athelicium hallenbergii ** |  |  |  |  |  |  |  |  |  |  |  |  |  |  | **3** | 1 | 2 |  |  |  |  | **3** |  |  |  |  |  |  |  |
| *Athelopsis lembospora* | **1** |  |  |  | 1 |  |  |  |  |  |  |  |  |  |  |  |  |  |  |  |  | **1** |  |  |  |  |  |  |  |
| *Athelopsis subinsconspicua (coll.)* |  |  |  |  |  |  |  |  |  |  |  |  |  |  | **1** |  |  | 1 |  |  |  | **1** |  |  |  |  |  |  |  |
| *Basidiodendron caesiocinereum* |  |  |  |  |  |  |  | **2** |  |  |  |  | 1 | 1 |  |  |  |  |  |  |  | **2** |  |  |  |  |  |  |  |
| *Basidiodendron cinereum* | **2** |  |  | 1 | 1 |  |  |  |  |  |  |  |  |  |  |  |  |  |  |  |  | **2** |  |  |  |  |  |  |  |
| *Basidiodendron eyrei* | **8** |  |  | 1 | 3 | 4 |  |  |  |  |  |  |  |  | **1** |  |  |  | 1 |  |  | **9** |  |  |  |  |  |  |  |
| *Basidioradulum radula* | **3** |  |  |  | 2 |  | 1 | **1** |  |  |  | 1 |  |  | **4** |  |  | 1 | 1 | 2 |  | **8** |  |  |  |  |  |  |  |
| *Bjerkandera adusta* | **2** |  |  |  |  |  | 2 |  |  |  |  |  |  |  | **2** |  |  |  |  |  | 2 | **4** |  |  |  |  |  |  |  |
| *Boidinia sp. (nova)* |  |  |  |  |  |  |  |  |  |  |  |  |  |  | **3** |  | 3 |  |  |  |  | **3** |  |  |  |  |  |  |  |
| *Botryobasidium aureum ** | **3** |  |  |  | 1 | 2 |  |  |  |  |  |  |  |  |  |  |  |  |  |  |  | **3** |  |  |  |  |  |  |  |
| *Botryobasidium isabellinus* | **2** |  |  | 1 | 1 |  |  |  |  |  |  |  |  |  | **1** |  | 1 |  |  |  |  | **3** |  |  |  |  |  |  |  |
| *Botryobasidium laeve* | **22** | 3 | 6 | 2 | 5 |  | 6 |  |  |  |  |  |  |  |  |  |  |  |  |  |  | **22** |  |  |  |  |  |  |  |
| *Botryobasidium subcoronatum* | **1** |  |  |  |  | 1 |  | **1** |  |  |  |  | 1 |  |  |  |  |  |  |  |  | **2** |  |  |  |  |  |  |  |
| *Brevicellium olivascens* | **5** |  |  | 2 | 2 | 1 |  |  |  |  |  |  |  |  | **2** |  |  | 2 |  |  |  | **7** |  |  |  |  |  |  |  |
| *Byssomerulius corium* | **1** |  |  |  | 1 |  |  |  |  |  |  |  |  |  |  |  |  |  |  |  |  | **1** |  |  |  |  |  |  |  |
| *Byssomerulius jose-ferreirae ** | **3** |  | 1 |  |  | 1 | 1 |  |  |  |  |  |  |  | **1** |  | 1 |  |  |  |  | **4** |  |  |  |  |  |  |  |
| *Ceraceomyces serpens* | **1** |  |  |  | 1 |  |  |  |  |  |  |  |  |  | **2** |  | 2 |  |  |  |  | **3** |  |  |  |  |  |  |  |
| *Ceraceomyces sp. (nova)* | **1** |  |  |  | 1 |  |  |  |  |  |  |  |  |  |  |  |  |  |  |  |  | **1** |  |  |  |  |  |  |  |
| *Ceraceomyces tessulatus* |  |  |  |  |  |  |  | **1** | 1 |  |  |  |  |  |  |  |  |  |  |  |  | **1** |  |  |  |  |  |  |  |
| *Ceratobasidium aff.cornigerum* |  |  |  |  |  |  |  |  |  |  |  |  |  |  | **1** | 1 |  |  |  |  |  | **1** |  |  |  |  |  |  |  |
| *Ceratobasidium cornigerum* | **4** |  | 3 | 1 |  |  |  | **2** | 1 | 1 |  |  |  |  | **3** | 2 | 1 |  |  |  |  | **9** |  |  |  |  |  |  |  |
| *Ceriporia pseudogilvescens* |  |  |  |  |  |  |  |  |  |  |  |  |  |  | **1** |  |  |  |  | 1 |  | **1** |  |  |  |  |  |  |  |
| *Ceriporia reticulata* | **13** |  | 1 | 2 | 4 | 6 |  | **3** |  |  | 1 | 2 |  |  | **1** |  |  |  | 1 |  |  | **17** |  |  |  |  |  |  |  |
| *Cerrena unicolor* |  |  |  |  |  |  |  |  |  |  |  |  |  |  | **1** |  |  |  |  |  | 1 | **1** |  |  |  |  |  |  |  |
| *Chondrostereum purpureum* | **2** |  |  | 1 |  |  | 1 | **1** |  |  |  |  |  | 1 |  |  |  |  |  |  |  | **3** |  |  |  |  |  |  |  |
| *Colacogloea peniophorae* | **1** |  |  |  | 1 |  |  |  |  |  |  |  |  |  |  |  |  |  |  |  |  | **1** |  |  |  |  |  |  |  |
| *Conferticium ravum* (VU) |  |  |  |  |  |  |  | **2** | 2 |  |  |  |  |  |  |  |  |  |  |  |  | **2** |  |  |  |  |  |  |  |
| *Coniophora puteana* | **2** |  |  |  |  |  | 2 |  |  |  |  |  |  |  |  |  |  |  |  |  |  | **2** |  |  |  |  |  |  |  |
| *Coronicium alboglaucum ** |  |  |  |  |  |  |  | **3** | 3 |  |  |  |  |  |  |  |  |  |  |  |  | **3** |  |  |  |  |  |  |  |
| *Corticium roseum* | **1** |  |  |  |  | 1 |  | **1** |  |  |  | 1 |  |  | **2** |  |  |  | 2 |  |  | **4** |  |  |  |  |  |  |  |
| *Cristinia cf.rheana* | **1** |  |  |  |  | 1 |  |  |  |  |  |  |  |  |  |  |  |  |  |  |  | **1** |  |  |  |  |  |  |  |
| *Cristinia helvetica* |  |  |  |  |  |  |  | **1** | 1 |  |  |  |  |  |  |  |  |  |  |  |  | **1** |  |  |  |  |  |  |  |
| *Cristinia rheana *** |  |  |  |  |  |  |  | **1** |  |  |  |  | 1 |  |  |  |  |  |  |  |  | **1** |  |  |  |  |  |  |  |
| *Cylindrobasidium evolvens* | **13** |  | 2 |  | 4 | 3 | 4 | **3** | 2 |  |  | 1 |  |  | **2** | 1 |  |  | 1 |  |  | **18** |  |  |  |  |  |  |  |
| *Cytidia salicina* | **6** |  |  | 1 | 2 | 1 | 2 |  |  |  |  |  |  |  | **5** |  | 4 |  | 1 |  |  | **11** |  |  |  |  |  |  |  |
| *Datronia mollis* | **8** | 1 |  |  | 3 | 2 | 2 |  |  |  |  |  |  |  | **1** |  |  |  |  | 1 |  | **9** |  |  |  |  |  |  |  |
| *Dendrothele amygdalispora ** | **1** |  |  |  |  |  | 1 |  |  |  |  |  |  |  |  |  |  |  |  |  |  | **1** |  |  |  |  |  |  |  |
| *Dendrothele commixta* | **1** | 1 |  |  |  |  |  |  |  |  |  |  |  |  |  |  |  |  |  |  |  | **1** |  |  |  |  |  |  |  |
| *Fomes fomentarius* | **13** |  |  |  |  | 2 | 11 | **3** |  |  |  |  | 1 | 2 | **2** |  |  |  |  | 2 |  | **18** |  |  |  |  |  |  |  |
| *Fomitopsis pinicola* | **12** |  |  |  | 1 | 1 | 10 |  |  |  |  |  |  |  |  |  |  |  |  |  |  | **12** |  |  |  |  |  |  |  |
| *Galzinia incrustans* |  |  |  |  |  |  |  |  |  |  |  |  |  |  | **5** |  | 2 | 2 | 1 |  |  | **5** |  |  |  |  |  |  |  |
| *Globulicium hiemale* | **1** |  |  |  | 1 |  |  |  |  |  |  |  |  |  |  |  |  |  |  |  |  | **1** |  |  |  |  |  |  |  |
| *Gloeocystidiellum porosum* | **2** |  |  | 1 |  |  | 1 | **3** |  | 1 | 1 | 1 |  |  | **14** | 3 | 5 | 2 | 4 |  |  | **19** |  |  |  |  |  |  |  |
| *Gloeopeniophorella convolvens* | **3** |  | 1 | 1 |  |  | 1 |  |  |  |  |  |  |  | **1** |  |  | 1 |  |  |  | **4** |  |  |  |  |  |  |  |
| *Gloeophyllum sepiarinum* |  |  |  |  |  |  |  | **1** |  |  |  |  |  | 1 |  |  |  |  |  |  |  | **1** |  |  |  |  |  |  |  |
| *Gloeoporus dichrous* | **1** |  |  |  |  |  | 1 |  |  |  |  |  |  |  |  |  |  |  |  |  |  | **1** |  |  |  |  |  |  |  |
| *Gyrophanopsis polonensis* | **1** |  |  |  |  | 1 |  |  |  |  |  |  |  |  |  |  |  |  |  |  |  | **1** |  |  |  |  |  |  |  |
| *Hapalopilus rutilans* |  |  |  |  |  |  |  |  |  |  |  |  |  |  | **1** |  | 1 |  |  |  |  | **1** |  |  |  |  |  |  |  |
| *Hymenochaete cinnamomea* | **2** |  |  |  | 2 |  |  |  |  |  |  |  |  |  |  |  |  |  |  |  |  | **2** |  |  |  |  |  |  |  |
| *Hymenochaete tabacina* | **2** |  |  | 1 |  |  | 1 |  |  |  |  |  |  |  | **20** | 7 | 5 |  | 7 | 1 |  | **22** |  |  |  |  |  |  |  |
| *Hyphoderma argillaceum* | **1** |  |  |  |  | 1 |  |  |  |  |  |  |  |  |  |  |  |  |  |  |  | **1** |  |  |  |  |  |  |  |
| *Hyphoderma cf.roseocremeum* |  |  |  |  |  |  |  |  |  |  |  |  |  |  | **1** |  |  |  | 1 |  |  | **1** |  |  |  |  |  |  |  |
| *Hyphoderma incrustatum* (DD) *** | **1** |  |  |  |  |  | 1 |  |  |  |  |  |  |  |  |  |  |  |  |  |  | **1** |  |  |  |  |  |  |  |
| *Hyphoderma medioburiense* | **1** |  |  |  | 1 |  |  | **1** |  |  |  | 1 |  |  |  |  |  |  |  |  |  | **2** |  |  |  |  |  |  |  |
| *Hyphoderma nemorale* |  |  |  |  |  |  |  |  |  |  |  |  |  |  | **2** |  |  |  | 2 |  |  | **2** |  |  |  |  |  |  |  |
| *Hyphoderma occidentale* | **1** |  |  |  | 1 |  |  |  |  |  |  |  |  |  |  |  |  |  |  |  |  | **1** |  |  |  |  |  |  |  |
| *Hyphoderma setigerum* | **8** |  |  |  | 6 |  | 2 | **8** |  |  | 2 | 5 | 1 |  | **13** | 1 | 4 | 2 | 6 |  |  | **29** |  |  |  |  |  |  |  |
| *Hyphodontia alutaria* | **1** |  |  |  | 1 |  |  |  |  |  |  |  |  |  |  |  |  |  |  |  |  | **1** |  |  |  |  |  |  |  |
| *Hyphodontiella hauerslevii *** | **1** |  | 1 |  |  |  |  |  |  |  |  |  |  |  | **1** | 1 |  |  |  |  |  | **2** |  |  |  |  |  |  |  |
| *Hyphodontiella multiseptata* | **1** |  |  | 1 |  |  |  |  |  |  |  |  |  |  |  |  |  |  |  |  |  | **1** |  |  |  |  |  |  |  |
| *Hypochnicium bombycinum* | **3** |  |  |  |  | 1 | 2 |  |  |  |  |  |  |  |  |  |  |  |  |  |  | **3** |  |  |  |  |  |  |  |
| *Hypochnicium punctulatum* |  |  |  |  |  |  |  |  |  |  |  |  |  |  | **2** | 1 |  | 1 |  |  |  | **2** |  |  |  |  |  |  |  |
| *Irpex oreophilus* |  |  |  |  |  |  |  | **2** | 2 |  |  |  |  |  |  |  |  |  |  |  |  | **2** |  |  |  |  |  |  |  |
| *Junghuhnia nitida* | **16** |  | 4 | 2 | 10 |  |  | **2** |  |  | 1 | 1 |  |  | **1** |  |  |  | 1 |  |  | **19** |  |  |  |  |  |  |  |
| *Kneiffiella barba-jovis* | **4** |  |  |  | 1 | 3 |  | **2** |  |  |  | 2 |  |  |  |  |  |  |  |  |  | **6** |  |  |  |  |  |  |  |
| *Kneiffiella subalutacea* | **5** |  |  |  | 1 | 3 | 1 | **1** |  |  | 1 |  |  |  |  |  |  |  |  |  |  | **6** |  |  |  |  |  |  |  |
| *Lagarobasidium detritica* | **7** |  | 2 | 3 | 1 |  | 1 |  |  |  |  |  |  |  | **4** | 3 | 1 |  |  |  |  | **11** |  |  |  |  |  |  |  |
| *Leptosporomyces aff.fusoideus* |  |  |  |  |  |  |  | **3** | 2 | 1 |  |  |  |  |  |  |  |  |  |  |  | **3** |  |  |  |  |  |  |  |
| *Leptosporomyces fusoideus* |  |  |  |  |  |  |  |  |  |  |  |  |  |  | **1** | 1 |  |  |  |  |  | **1** |  |  |  |  |  |  |  |
| *Leptosporomyces galzinii* |  |  |  |  |  |  |  | **12** | 8 | 1 | 1 | 2 |  |  |  |  |  |  |  |  |  | **12** |  |  |  |  |  |  |  |
| *Leptosporomyces montanus (cf.)* | **3** |  |  | 3 |  |  |  |  |  |  |  |  |  |  | **4** |  | 2 | 2 |  |  |  | **7** |  |  |  |  |  |  |  |
| *Litschauerella clematitis ** | **1** |  | 1 |  |  |  |  |  |  |  |  |  |  |  |  |  |  |  |  |  |  | **1** |  |  |  |  |  |  |  |
| *Lyomyces erastii* | **11** | 3 | 2 | 4 | 1 |  | 1 |  |  |  |  |  |  |  | **2** | 2 |  |  |  |  |  | **13** |  |  |  |  |  |  |  |
| *Lyomyces incrustatus* | **2** |  |  | 2 |  |  |  |  |  |  |  |  |  |  |  |  |  |  |  |  |  | **2** |  |  |  |  |  |  |  |
| *Lyomyces sambuci* | **40** | 1 | 8 | 11 | 13 | 2 | 5 |  |  |  |  |  |  |  | **9** | 9 |  |  |  |  |  | **49** |  |  |  |  |  |  |  |
| *Merismodes fasciculata* | **1** |  |  | 1 |  |  |  | **11** | 2 | 3 | 1 | 4 | 1 |  | **135** | 18 | 66 | 25 | 26 |  |  | **147** |  |  |  |  |  |  |  |
| *Mucronella calva* | **2** |  |  |  |  | 2 |  |  |  |  |  |  |  |  |  |  |  |  |  |  |  | **2** |  |  |  |  |  |  |  |
| *Mycoacia aurea* | **4** |  |  |  | 2 | 1 | 1 |  |  |  |  |  |  |  |  |  |  |  |  |  |  | **4** |  |  |  |  |  |  |  |
| *Mycoacia fuscoatra* |  |  |  |  |  |  |  | **1** |  |  |  | 1 |  |  |  |  |  |  |  |  |  | **1** |  |  |  |  |  |  |  |
| *Odonticium flabelliradiatum ** |  |  |  |  |  |  |  |  |  |  |  |  |  |  | **5** | 1 | 2 |  | 1 | 1 |  | **5** |  |  |  |  |  |  |  |
| *Oliveonia fibrillosa ** |  |  |  |  |  |  |  | **1** |  |  |  | 1 |  |  |  |  |  |  |  |  |  | **1** |  |  |  |  |  |  |  |
| *Oliveonia pauxilla ** |  |  |  |  |  |  |  |  |  |  |  |  |  |  | **1** |  |  | 1 |  |  |  | **1** |  |  |  |  |  |  |  |
| *Oliveonia sp. (nova)** | **2** |  |  | 1 |  |  | 1 |  |  |  |  |  |  |  |  |  |  |  |  |  |  | **2** |  |  |  |  |  |  |  |
| *aff. Oliveonia (sp.nova)* |  |  |  |  |  |  |  |  |  |  |  |  |  |  | **1** |  | 1 |  |  |  |  | **1** |  |  |  |  |  |  |  |
| *Peniophora cinerea* | **8** |  | 3 | 3 | 2 |  |  |  |  |  |  |  |  |  | **7** | 7 |  |  |  |  |  | **15** |  |  |  |  |  |  |  |
| *Peniophora incarnata* | **9** | 1 | 1 | 2 | 2 | 1 | 2 | **9** | 4 | 1 |  | 3 | 1 |  | **15** | 11 | 1 | 1 | 2 |  |  | **33** |  |  |  |  |  |  |  |
| *Peniophora nuda* | **10** | 2 | 5 |  | 2 |  | 1 | **2** | 1 | 1 |  |  |  |  | **6** | 5 | 1 |  |  |  |  | **18** |  |  |  |  |  |  |  |
| *Peniophora pithya* | **2** |  |  |  |  |  | 2 |  |  |  |  |  |  |  |  |  |  |  |  |  |  | **2** |  |  |  |  |  |  |  |
| *Peniophora violaceolivida* | **2** |  | 1 |  |  |  | 1 |  |  |  |  |  |  |  |  |  |  |  |  |  |  | **2** |  |  |  |  |  |  |  |
| *Peniophorella pallida* | **1** |  |  |  | 1 |  |  |  |  |  |  |  |  |  |  |  |  |  |  |  |  | **1** |  |  |  |  |  |  |  |
| *Peniophorella praetermissa* | **19** |  | 1 | 4 | 9 |  | 5 | **3** |  |  |  | 2 | 1 |  | **7** |  | 3 |  | 3 | 1 |  | **29** |  |  |  |  |  |  |  |
| *Peniophorella pubera* | **4** |  |  |  |  | 1 | 3 | **3** |  |  |  | 1 |  | 2 |  |  |  |  |  |  |  | **7** |  |  |  |  |  |  |  |
| *Phanerochaete laevis* |  |  |  |  |  |  |  | **8** |  | 2 | 3 | 3 |  |  | **2** | 1 |  | 1 |  |  |  | **10** |  |  |  |  |  |  |  |
| *Phanerochaete sanguinea* | **55** |  | 4 | 5 | 27 | 14 | 5 |  |  |  |  |  |  |  |  |  |  |  |  |  |  | **55** |  |  |  |  |  |  |  |
| *Phanerochaete sordida* | **2** |  |  |  | 2 |  |  | **2** |  |  | 1 |  |  | 1 | **11** | 6 | 3 | 2 |  |  |  | **15** |  |  |  |  |  |  |  |
| *Phanerochaete tuberculata* | **6** |  |  |  | 3 | 1 | 2 |  |  |  |  |  |  |  |  |  |  |  |  |  |  | **6** |  |  |  |  |  |  |  |
| *Phanerochaete velutina* | **1** |  |  |  |  |  | 1 | **3** | 2 |  |  |  | 1 |  | **15** | 9 | 1 | 2 | 2 | 1 |  | **19** |  |  |  |  |  |  |  |
| *Phellinus cinereus* | **1** |  |  |  |  |  | 1 | **1** |  |  |  |  |  | 1 | **1** |  |  |  |  |  | 1 | **3** |  |  |  |  |  |  |  |
| *Phellinus conchatus* | **2** |  |  |  |  |  | 2 |  |  |  |  |  |  |  |  |  |  |  |  |  |  | **2** |  |  |  |  |  |  |  |
| *Phellinus laevigatus* |  |  |  |  |  |  |  | **1** |  |  |  | 1 |  |  |  |  |  |  |  |  |  | **1** |  |  |  |  |  |  |  |
| *Phellinus punctatus* | **1** |  |  |  |  |  | 1 |  |  |  |  |  |  |  |  |  |  |  |  |  |  | **1** |  |  |  |  |  |  |  |
| *Phellinus tremulae* | **1** |  |  |  |  |  | 1 |  |  |  |  |  |  |  |  |  |  |  |  |  |  | **1** |  |  |  |  |  |  |  |
| *Phlebia albida* | **1** |  |  |  |  |  | 1 |  |  |  |  |  |  |  |  |  |  |  |  |  |  | **1** |  |  |  |  |  |  |  |
| *Phlebia deflectens* |  |  |  |  |  |  |  | **2** |  |  |  | 2 |  |  |  |  |  |  |  |  |  | **2** |  |  |  |  |  |  |  |
| *Phlebia lilascens coll.* | **1** |  |  | 1 |  |  |  | **1** |  |  |  | 1 |  |  |  |  |  |  |  |  |  | **2** |  |  |  |  |  |  |  |
| *Phlebia nitidula* |  |  |  |  |  |  |  | **1** | 1 |  |  |  |  |  | **1** |  |  |  | 1 |  |  | **2** |  |  |  |  |  |  |  |
| *Phlebia radiata* | **7** |  |  |  | 1 | 1 | 5 |  |  |  |  |  |  |  | **1** |  |  |  | 1 |  |  | **8** |  |  |  |  |  |  |  |
| *Phlebia rufa* | **3** |  |  |  | 1 | 2 |  | **1** |  |  |  |  | 1 |  |  |  |  |  |  |  |  | **4** |  |  |  |  |  |  |  |
| *Phlebiella aff.insperata* |  |  |  |  |  |  |  |  |  |  |  |  |  |  | **134** | 89 | 31 | 8 | 6 |  |  | **134** |  |  |  |  |  |  |  |
| *Phlebiella borealis* |  |  |  |  |  |  |  |  |  |  |  |  |  |  | **1** |  |  | 1 |  |  |  | **1** |  |  |  |  |  |  |  |
| *Phlebiella insperata* (EN)*** | **1** |  |  |  | 1 |  |  |  |  |  |  |  |  |  |  |  |  |  |  |  |  | **1** |  |  |  |  |  |  |  |
| *Phlebiella sulphurea s.lato* | **1** |  |  |  | 1 |  |  | **1** |  |  |  |  |  | 1 | **1** |  | 1 |  |  |  |  | **3** |  |  |  |  |  |  |  |
| *Phlebiella tulasnelloidea* | **3** |  |  |  | 3 |  |  |  |  |  |  |  |  |  |  |  |  |  |  |  |  | **3** |  |  |  |  |  |  |  |
| *Piloderma byssinum* |  |  |  |  |  |  |  | **1** |  |  |  | 1 |  |  |  |  |  |  |  |  |  | **1** |  |  |  |  |  |  |  |
| *Piloderma fallax* | **9** |  | 1 |  | 3 | 5 |  | **18** | 12 | 6 |  |  |  |  |  |  |  |  |  |  |  | **27** |  |  |  |  |  |  |  |
| *Piloderma lanatum ** |  |  |  |  |  |  |  | **2** |  |  |  | 1 |  | 1 |  |  |  |  |  |  |  | **2** |  |  |  |  |  |  |  |
| *Piptoporus betulinus* |  |  |  |  |  |  |  | **2** |  |  |  | 1 | 1 |  | **1** |  |  |  | 1 |  |  | **3** |  |  |  |  |  |  |  |
| *Plicatura nivea* | **5** |  |  |  | 2 | 1 | 2 |  |  |  |  |  |  |  |  |  |  |  |  |  |  | **5** |  |  |  |  |  |  |  |
| *Polyporus brumalis* | **1** |  |  |  | 1 |  |  |  |  |  |  |  |  |  | **2** |  | 1 | 1 |  |  |  | **3** |  |  |  |  |  |  |  |
| *Polyporus ciliatus* |  |  |  |  |  |  |  |  |  |  |  |  |  |  | **1** |  |  | 1 |  |  |  | **1** |  |  |  |  |  |  |  |
| *Polyporus leptocephalus* | **3** |  |  |  | 3 |  |  |  |  |  |  |  |  |  |  |  |  |  |  |  |  | **3** |  |  |  |  |  |  |  |
| *Porotheleum fimbriatum* | **6** |  |  |  | 4 | 2 |  |  |  |  |  |  |  |  | **1** |  | 1 |  |  |  |  | **7** |  |  |  |  |  |  |  |
| *Postia alni* | **6** |  |  |  | 4 | 2 |  |  |  |  |  |  |  |  |  |  |  |  |  |  |  | **6** |  |  |  |  |  |  |  |
| *Radulomyces confluens* | **13** |  | 5 | 1 | 5 | 2 |  |  |  |  |  |  |  |  |  |  |  |  |  |  |  | **13** |  |  |  |  |  |  |  |
| *Ramaricium alboochraceum* (VU)* |  |  |  |  |  |  |  |  |  |  |  |  |  |  | **6** | 4 |  | 2 |  |  |  | **6** |  |  |  |  |  |  |  |
| *"Renatobasidium" (sp.nova)* |  |  |  |  |  |  |  | **1** |  |  |  | 1 |  |  |  |  |  |  |  |  |  | **1** |  |  |  |  |  |  |  |
| *Schizopora paradoxa* |  |  |  |  |  |  |  |  |  |  |  |  |  |  | **5** |  |  | 2 | 2 | 1 |  | **5** |  |  |  |  |  |  |  |
| *Scopuloides rimosa* | **11** |  | 2 | 3 | 5 |  | 1 |  |  |  |  |  |  |  | **5** |  | 2 | 1 | 1 | 1 |  | **16** |  |  |  |  |  |  |  |
| *Sebacina epigaea ** | **1** |  |  |  |  | 1 |  |  |  |  |  |  |  |  |  |  |  |  |  |  |  | **1** |  |  |  |  |  |  |  |
| *Sebacina helvelloides ** | **1** | 1 |  |  |  |  |  |  |  |  |  |  |  |  |  |  |  |  |  |  |  | **1** |  |  |  |  |  |  |  |
| *Sebacina incrustans* | **10** | 3 | 3 | 3 | 1 |  |  |  |  |  |  |  |  |  |  |  |  |  |  |  |  | **10** |  |  |  |  |  |  |  |
| *Sistotrema aff.binucleosporum* |  |  |  |  |  |  |  |  |  |  |  |  |  |  | **1** |  |  |  | 1 |  |  | **1** |  |  |  |  |  |  |  |
| *Sistotrema autumnale ** |  |  |  |  |  |  |  |  |  |  |  |  |  |  | **1** |  |  | 1 |  |  |  | **1** |  |  |  |  |  |  |  |
| *Sistotrema brinkmannii* | **15** | 2 | 3 | 1 | 4 |  | 5 | **5** |  |  | 1 | 3 | 1 |  | **11** | 1 | 2 | 2 | 3 | 3 |  | **31** |  |  |  |  |  |  |  |
| *Sistotrema cf.brinkmannii* | **1** |  |  | 1 |  |  |  | **1** |  |  |  | 1 |  |  |  |  |  |  |  |  |  | **2** |  |  |  |  |  |  |  |
| *Sistotrema cf.oblongisporum* | **3** | 2 | 1 |  |  |  |  |  |  |  |  |  |  |  | **9** |  | 2 |  | 7 |  |  | **12** |  |  |  |  |  |  |  |
| *Sistotrema diademiferum ** | **1** |  |  |  |  |  | 1 |  |  |  |  |  |  |  | **3** | 1 | 1 |  | 1 |  |  | **4** |  |  |  |  |  |  |  |
| *Sistotrema efibulatum ** |  |  |  |  |  |  |  | **6** | 6 |  |  |  |  |  |  |  |  |  |  |  |  | **6** |  |  |  |  |  |  |  |
| *Sistotrema muscicola* | **1** |  | 1 |  |  |  |  | **3** |  |  | 2 | 1 |  |  | **4** | 2 | 2 |  |  |  |  | **8** |  |  |  |  |  |  |  |
| *Sistotrema oblongisporum* | **1** |  | 1 |  |  |  |  | **6** | 1 | 4 |  | 1 |  |  | **16** | 1 | 6 | 3 | 6 |  |  | **23** |  |  |  |  |  |  |  |
| *Sistotrema octosporum* | **2** | 1 |  |  |  | 1 |  | **11** | 2 | 3 | 2 | 4 |  |  | **12** | 3 | 3 |  | 6 |  |  | **25** |  |  |  |  |  |  |  |
| *Sistotrema sernanderi* | **5** |  |  |  |  |  | 5 |  |  |  |  |  |  |  |  |  |  |  |  |  |  | **5** |  |  |  |  |  |  |  |
| *Sistotremastrum niveocremeum* | **2** |  | 1 |  | 1 |  |  | **1** |  |  |  | 1 |  |  | **3** | 2 |  | 1 |  |  |  | **6** |  |  |  |  |  |  |  |
| *Skeletocutis nivea coll.* | **2** |  |  | 1 | 1 |  |  |  |  |  |  |  |  |  |  |  |  |  |  |  |  | **2** |  |  |  |  |  |  |  |
| *Steccherinum fimbriatum* | **154** | 1 | 57 | 45 | 46 | 2 | 3 | **35** | 15 | 8 | 11 | 1 |  |  | **135** | 59 | 44 | 20 | 11 | 1 |  | **324** |  |  |  |  |  |  |  |
| *Steccherinum ochraceum coll.* | **19** | 3 | 7 | 6 | 3 |  |  | **3** | 1 | 2 |  |  |  |  | **186** | 115 | 66 | 3 | 1 | 1 |  | **208** |  |  |  |  |  |  |  |
| *Stereum hirsutum* | **2** |  |  |  | 2 |  |  | **3** |  |  |  | 2 | 1 |  | **3** | 1 |  |  | 1 | 1 |  | **8** |  |  |  |  |  |  |  |
| *Stereum rugosum* | **12** | 1 |  | 2 | 5 | 3 | 1 | **3** |  |  |  | 2 | 1 |  | **7** | 1 | 4 |  | 1 |  | 1 | **22** |  |  |  |  |  |  |  |
| *Stereum sanguinolentum* |  |  |  |  |  |  |  | **2** |  |  |  | 2 |  |  |  |  |  |  |  |  |  | **2** |  |  |  |  |  |  |  |
| *Stypella subgelatinosa ** |  |  |  |  |  |  |  |  |  |  |  |  |  |  | **2** |  |  |  | 2 |  |  | **2** |  |  |  |  |  |  |  |
| *Subulicystidium longisporum coll.* | **19** |  | 3 | 5 | 10 |  | 1 | **1** |  |  |  |  |  | 1 | **10** | 6 | 3 | 1 |  |  |  | **30** |  |  |  |  |  |  |  |
| *Thanatephorus fusisporus* | **1** |  |  |  |  |  | 1 | **2** |  |  |  | 1 | 1 |  | **1** |  | 1 |  |  |  |  | **4** |  |  |  |  |  |  |  |
| *Tomentella crinalis* | **5** |  |  |  | 3 |  | 2 |  |  |  |  |  |  |  |  |  |  |  |  |  |  | **5** |  |  |  |  |  |  |  |
| *Tomentella galzinii* |  |  |  |  |  |  |  |  |  |  |  |  |  |  | **45** | 41 | 4 |  |  |  |  | **45** |  |  |  |  |  |  |  |
| *Tomentella subclavigera ** |  |  |  |  |  |  |  |  |  |  |  |  |  |  | **3** | 2 | 1 |  |  |  |  | **3** |  |  |  |  |  |  |  |
| *Tomentellopsis echinospora* | **8** |  | 4 | 2 | 1 | 1 |  | **4** | 1 |  | 2 |  |  | 1 | **4** |  | 4 |  |  |  |  | **16** |  |  |  |  |  |  |  |
| *Trametes betulinus* |  |  |  |  |  |  |  |  |  |  |  |  |  |  | **1** |  |  |  |  | 1 |  | **1** |  |  |  |  |  |  |  |
| *Trametes ochracea* | **1** |  |  |  |  | 1 |  |  |  |  |  |  |  |  | **1** |  |  |  |  |  | 1 | **2** |  |  |  |  |  |  |  |
| *Trechispora byssinella* |  |  |  |  |  |  |  | **2** | 1 |  |  | 1 |  |  |  |  |  |  |  |  |  | **2** |  |  |  |  |  |  |  |
| *Trechispora cohaerens* | **1** |  | 1 |  |  |  |  |  |  |  |  |  |  |  | **15** | 12 | 2 |  | 1 |  |  | **16** |  |  |  |  |  |  |  |
| *Trechispora farinacea* | **7** |  |  |  | 1 | 5 | 1 | **4** | 1 |  |  | 2 |  | 1 | **3** | 1 | 1 |  | 1 |  |  | **14** |  |  |  |  |  |  |  |
| *Trechispora praefocata* | **13** | 4 | 6 | 2 | 1 |  |  |  |  |  |  |  |  |  |  |  |  |  |  |  |  | **13** |  |  |  |  |  |  |  |
| *Trechispora stellulata* |  |  |  |  |  |  |  |  |  |  |  |  |  |  | **1** |  | 1 |  |  |  |  | **1** |  |  |  |  |  |  |  |
| *Trechispora stevensoni* | **1** |  |  |  | 1 |  |  |  |  |  |  |  |  |  |  |  |  |  |  |  |  | **1** |  |  |  |  |  |  |  |
| *Trechispora subsphaeospora* | **1** |  |  | 1 |  |  |  |  |  |  |  |  |  |  |  |  |  |  |  |  |  | **1** |  |  |  |  |  |  |  |
| *Trechispora tenuicula* |  |  |  |  |  |  |  |  |  |  |  |  |  |  | **1** | 1 |  |  |  |  |  | **1** |  |  |  |  |  |  |  |
| *Trichaptum abietinum* | **1** |  |  |  |  |  | 1 |  |  |  |  |  |  |  |  |  |  |  |  |  |  | **1** |  |  |  |  |  |  |  |
| *Trichaptum fuscoviolaceum* | **1** |  |  |  |  |  | 1 |  |  |  |  |  |  |  |  |  |  |  |  |  |  | **1** |  |  |  |  |  |  |  |
| *Tubulicrinis glebulosus* | **2** |  |  |  | 2 |  |  |  |  |  |  |  |  |  | **70** | 12 | 37 | 9 | 12 |  |  | **72** |  |  |  |  |  |  |  |
| *Tubulicrinis sororius* | **1** |  |  |  |  | 1 |  | **1** |  |  |  | 1 |  |  |  |  |  |  |  |  |  | **2** |  |  |  |  |  |  |  |
| *Tubulicrinis subulatus* | **6** |  |  | 1 | 2 | 1 | 2 |  |  |  |  |  |  |  | **1** |  | 1 |  |  |  |  | **7** |  |  |  |  |  |  |  |
| *Tulasnella albida* | **2** |  |  |  | 2 |  |  | **3** |  |  | 1 | 2 |  |  | **13** | 1 | 2 | 8 | 1 | 1 |  | **18** |  |  |  |  |  |  |  |
| *Tulasnella eichleriana* |  |  |  |  |  |  |  | **1** |  |  |  | 1 |  |  | **1** |  |  | 1 |  |  |  | **2** |  |  |  |  |  |  |  |
| *Tulasnella violea* | **2** |  |  |  |  | 1 | 1 | **2** |  |  |  | 2 |  |  |  |  |  |  |  |  |  | **4** |  |  |  |  |  |  |  |
| *Tylospora asterophora* | **7** | 2 | 1 | 3 | 1 |  |  | **5** |  | 2 | 2 | 1 |  |  | **40** | 20 | 15 | 5 |  |  |  | **52** |  |  |  |  |  |  |  |
| *Vuilleminia (cf.) erastii* |  |  |  |  |  |  |  | **1** |  |  | 1 |  |  |  |  |  |  |  |  |  |  | **1** |  |  |  |  |  |  |  |
| *Vuilleminia comedens* | **2** |  |  | 1 |  |  | 1 |  |  |  |  |  |  |  |  |  |  |  |  |  |  | **2** |  |  |  |  |  |  |  |
| *Xenasma pruinosum *** | **4** |  |  | 3 |  | 1 |  |  |  |  |  |  |  |  |  |  |  |  |  |  |  | **4** |  |  |  |  |  |  |  |
| *Xylodon aff. brevisetus* | **1** |  |  | 1 |  |  |  |  |  |  |  |  |  |  |  |  |  |  |  |  |  | **1** |  |  |  |  |  |  |  |
| *Xylodon borealis* |  |  |  |  |  |  |  | **10** |  | 3 | 2 | 5 |  |  | **67** | 43 | 19 | 4 | 1 |  |  | **77** |  |  |  |  |  |  |  |
| *Xylodon brevisetus* | **1** |  |  |  |  |  | 1 |  |  |  |  |  |  |  |  |  |  |  |  |  |  | **1** |  |  |  |  |  |  |  |
| *Xylodon crustosus* | **29** | 1 | 2 | 6 | 18 | 2 |  | **1** |  |  | 1 |  |  |  | **12** | 1 | 5 | 1 | 4 | 1 |  | **42** |  |  |  |  |  |  |  |
| *Xylodon rimosissimus* | **23** |  | 1 | 6 | 8 | 4 | 4 | **1** |  | 1 |  |  |  |  |  |  |  |  |  |  |  | **24** |  |  |  |  |  |  |  |
| *Xylodon sp. "langerii"* |  |  |  |  |  |  |  | **1** |  |  |  | 1 |  |  |  |  |  |  |  |  |  | **1** |  |  |  |  |  |  |  |
| Total | **849** | 35 | 148 | 159 | 275 | 97 | 135 | **290** | 79 | 48 | 47 | 86 | 16 | 14 | **1399** | 641 | 423 | 143 | 159 | 27 | 6 | **2538** |  |  |  |  |  |  |  |
|  |  |  |  |  |  |  |  |  |  |  |  |  |  |  |  |  |  |  |  |  |  |  |  |  |  |  |  |  |  |
| **Genus level** |  |  |  |  |  |  |  |  |  |  |  |  |  |  |  |  |  |  |  |  |  |  |  |  |  |  |  |  |  |
|  |  |  |  |  |  |  |  |  |  |  |  |  |  |  |  |  |  |  |  |  |  |  |  |  |  |  |  |  |  |
| *Antrodiella sp.* |  |  |  |  |  |  |  |  |  |  |  |  |  |  | **2** |  | 1 | 1 |  |  |  | **2** |  |  |  |  |  |  |  |
| *Athelia sp.* |  |  |  |  |  |  |  |  |  |  |  |  |  |  | **1** |  | 1 |  |  |  |  | **1** |  |  |  |  |  |  |  |
| *Hyphoderma sp.* |  |  |  |  |  |  |  |  |  |  |  |  |  |  | **1** |  |  |  | 1 |  |  | **1** |  |  |  |  |  |  |  |
| *Hyphodontia sp.* |  |  |  |  |  |  |  |  |  |  |  |  |  |  | **1** |  | 1 |  |  |  |  | **1** |  |  |  |  |  |  |  |
| *Junghuhnia sp.* | **1** |  |  | 1 |  |  |  |  |  |  |  |  |  |  |  |  |  |  |  |  |  | **1** |  |  |  |  |  |  |  |
| *Peniophora sp.* | **2** | 1 |  |  |  |  | 1 |  |  |  |  |  |  |  |  |  |  |  |  |  |  | **2** |  |  |  |  |  |  |  |
| *Phanerochaete sp.* |  |  |  |  |  |  |  |  |  |  |  |  |  |  | **6** | 3 | 3 |  |  |  |  | **6** |  |  |  |  |  |  |  |
| *Phellinus sp.* | **1** |  |  |  |  | 1 |  |  |  |  |  |  |  |  |  |  |  |  |  |  |  | **1** |  |  |  |  |  |  |  |
| *Scytinostroma sp.* | **1** |  |  |  |  |  | 1 |  |  |  |  |  |  |  |  |  |  |  |  |  |  | **1** |  |  |  |  |  |  |  |
| *Tomentella spp.* | **83** | 5 | 21 | 25 | 19 | 6 | 7 | **46** | 18 | 11 | 3 | 11 | 1 | 2 | **424** | 237 | 143 | 34 | 9 | 1 |  | **553** |  |  |  |  |  |  |  |
| *Trametes sp.* |  |  |  |  |  |  |  |  |  |  |  |  |  |  | **1** |  |  |  |  |  | 1 | **1** |  |  |  |  |  |  |  |
| *Trechispora sp.* | **1** |  |  | 1 |  |  |  |  |  |  |  |  |  |  | **1** |  |  | 1 |  |  |  | **2** |  |  |  |  |  |  |  |
| *Vuilleminia sp.* |  |  |  |  |  |  |  |  |  |  |  |  |  |  | **1** |  |  |  | 1 |  |  | **1** |  |  |  |  |  |  |  |
| Total | **89** | 6 | 21 | 27 | 19 | 7 | 9 | **46** | 18 | 11 | 3 | 11 | 1 | 2 | **438** | 240 | 149 | 36 | 11 | 1 | 1 | **573** |  |  |  |  |  |  |  |
|  |  |  |  |  |  |  |  |  |  |  |  |  |  |  |  |  |  |  |  |  |  |  |  |  |  |  |  |  |  |
| **Higher level taxa** |  |  |  |  |  |  |  |  |  |  |  |  |  |  |  |  |  |  |  |  |  |  |  |  |  |  |  |  |  |
|  |  |  |  |  |  |  |  |  |  |  |  |  |  |  |  |  |  |  |  |  |  |  |  |  |  |  |  |  |  |
| *Basidiomycete spp.* | **122** | 26 | 43 | 23 | 19 | 5 | 6 | **680** | 405 | 187 | 39 | 45 | 2 | 2 | **3121** | 2103 | 808 | 169 | 39 | 2 |  | **3923** |  |  |  |  |  |  |  |
| *Heterobasidiomycete sp.* |  |  |  |  |  |  |  |  |  |  |  |  |  |  | **1** |  | 1 |  |  |  |  | **1** |  |  |  |  |  |  |  |
| *Polyporaceae sp.* | **1** |  |  |  | 1 |  |  |  |  |  |  |  |  |  |  |  |  |  |  |  |  | **1** |  |  |  |  |  |  |  |
| Total | **123** | 26 | 43 | 23 | 20 | 5 | 6 | **680** | 405 | 187 | 39 | 45 | 2 | 2 | **3122** | 2103 | 809 | 169 | 39 | 2 | 0 | **3925** |  |  |  |  |  |  |  |
|  |  |  |  |  |  |  |  |  |  |  |  |  |  |  |  |  |  |  |  |  |  |  |  |  |  |  |  |  |  |
| **Grand Total** | **1061** | **67** | **212** | **209** | **314** | **109** | **150** | **1016** | **502** | **246** | **89** | **142** | **19** | **18** | **4959** | **2984** | **1381** | **348** | **209** | **30** | **7** | **7036** |  |  |  |  |  |  |  |
|  |  |  |  |  |  |  |  |  |  |  |  |  |  |  |  |  |  |  |  |  |  |  |  |  |  |  |  |  |  |
|  |  |  |  |  |  |  |  |  |  |  |  |  |  |  |  |  |  |  |  |  |  |  |  |  |  |  |  |  |  |

**References**

Bernicchia, A. & Gorjón, S.P. (2010) *Corticiaceae s.l.* Italia, Candusso.

Kotiranta, H., Saarenoksa, R. & Kytövuori, I. (2009) Aphyllophoroid fungi of Finland. A check-list with ecology, distribution and threat categories. *Norrlinia,* **19**, 1-223.

Kotiranta, H., Junninen, K., Saarenoksa, R., Kinnunen, J. & Kytövuori, I. (2010) Aphylloporales & heterobasidiomycetes. *Suomen Lajien Uhanalaisuus - Punainen Kirja 2010* (eds P. Rassi, E. Hyvärinen, A. Juslen & I. Mannerkoski), pp. 249-263. Helsinki, Ympäristöministeriö & Suomen ympäristökeskus.

Kunttu, P., Kulju, M., Pennanen, J., Kotiranta, H. & Halme, P. (2011) Additions to the Finnish aphylloporoid fungi. *Folia Cryptogamica Estonica,* **48**, 25-25-30.

Kunttu, P., Kulju, M. & Kotiranta, H. (2012) New national and regional biological records for Finland 2. Contributions to the Finnish aphyllophoroid funga (basidiomycota). *Memoranda - Societatis Pro Fauna Et Flora Fennica,* **88**, 61-66.

Kunttu, P., Pennanen, J., Kulju, M., Kekki, T. & Suominen, M. (2014) Noteworthy records of aphyllophoroid fungi in Finland (basidiomycota). *Acta Mycologica,* **49**, 221-235.

Kunttu, P., Pennanen, J., Helo, T., Kulju, M. & Söderholm, U. (2013) New national and regional biological records for Finland 4. Additions to the knowledge of Finnish aphyllophoroid funga (basidiomycota). *Memoranda - Societatis Pro Fauna Et Flora Fennica,* **89**, 119-124.

Ryvarden, L. & Melo, I. (2014) *Poroid Fungi of Europe.* Synopsis Fungorum.
